# Insensitivity of machine log files to MLC leaf backlash and effect of MLC backlash on clinical dynamic MLC motion: An experimental investigation

**DOI:** 10.1002/acm2.13660

**Published:** 2022-06-09

**Authors:** Michael Barnes, Dennis Pomare, Marcus Doebrich, Therese S. Standen, Joshua Wolf, Peter Greer, John Simpson

**Affiliations:** ^1^ Department of Radiation Oncology Calvary Mater Hospital Newcastle Newcastle New South Wales Australia; ^2^ School of Mathematical and Physical Sciences University of Newcastle Newcastle New South Wales Australia; ^3^ Icon Cancer Centre Maitland Maitland Private Hospital Maitland New South Wales Australia

**Keywords:** EPID, log files, MLC, quality assurance (QA)

## Abstract

**Purpose:**

Multi‐leaf‐collimator (MLC) leaf position accuracy is important for accurate dynamic radiotherapy treatment plan delivery. Machine log files have become widely utilized for quality assurance (QA) of such dynamic treatments. The primary aim is to test the sensitivity of machine log files in comparison to electronic portal imaging device (EPID)‐based measurements to MLC position errors caused by leaf backlash. The secondary aim is to investigate the effect of MLC leaf backlash on MLC leaf motion during clinical dynamic plan delivery.

**Methods:**

The sensitivity of machine log files and two EPID‐based measurements were assessed via a controlled experiment, whereby the length of the “T” section of a series of 12 MLC leaf T‐nuts in a Varian Millennium MLC for a Trilogy C‐series type linac was reduced by sandpapering the top of the “T” to introduce backlash. The built‐in machine MLC leaf backlash test as well as measurements for two EPID‐based dynamic MLC positional tests along with log files were recorded pre‐ and post‐T‐nut modification. All methods were investigated for sensitivity to the T‐nut change by assessing the effect on measured MLC leaf positions. A reduced version of the experiment was repeated on a TrueBeam type linac with Millennium MLC.

**Results:**

No significant differences before and after T‐nut modification were detected in any of the log file data. Both EPID methods demonstrated sensitivity to the introduced change at approximately the expected magnitude with a strong dependence observed with gantry angle. EPID‐based data showed MLC positional error in agreement with the micrometer measured T‐nut length change to 0.07 ± 0.05 mm (1 SD) using the departmental routine QA test. Backlash results were consistent between linac types.

**Conclusion:**

Machine log files appear insensitive to MLC position errors caused by MLC leaf backlash introduced via the T‐nut. The effect of backlash on clinical MLC motions is heavily gantry angle dependent.

## 1 INTRODUCTION

Maintaining multi‐leaf‐collimator (MLC) leaf position accuracy has been demonstrated to be important for accurate dynamic radiotherapy treatment delivery.^1^, ^2^, ^3^, ^4^, ^5^, ^6^, ^7^, ^8^, ^9^, ^10^ To mitigate the risk of MLC position and other treatment delivery errors, patient‐specific and routine quality assurance (QA) testing has been recommended.^11^, ^12^, ^13^, ^14^, ^15^, ^16^, ^17^ For routine MLC position QA, a tolerance of 1 mm^12^, ^16^, ^17^ has usually been prescribed, although for dynamic leaf position accuracy the tolerance has been recommended to be 0.5 mm.^17^

**FIGURE 1:**
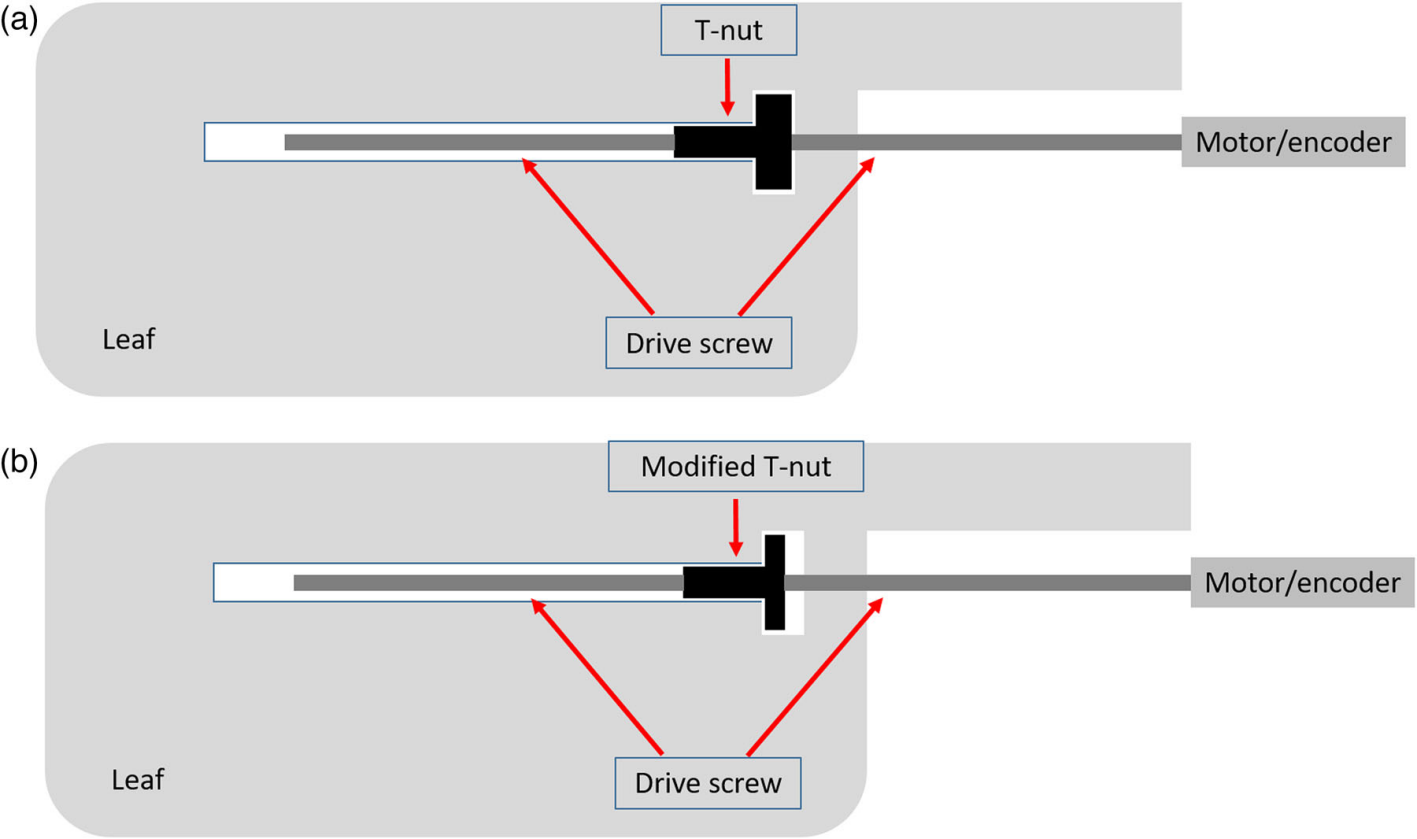
Schematic diagram of a Varian multi‐leaf‐collimator (MLC) leaf showing the mechanical relationship between the MLC motor, lead screw, T‐nut, and MLC leaf (not to scale). (a) Without modification, (b) after modification via sandpapering the top of the “T” as performed in this study.

For Varian (Varian Medical Systems, Palo Alto, CA, USA), MLCs, leaves are moved via an MLC motor/encoder for which the number of counts sets the leaf position. Via a coupler, the MLC motor/encoder turns a threaded drive screw into a plastic T‐nut, which is also threaded and hence translates as the screw rotates. The T‐nut is housed within the MLC leaf so that when the nut is translated it pushes (or pulls) the MLC leaf along its track between other leaves. As such, the T‐nut provides the mechanical connection between the drive screw and MLC leaf (Figure 1). In the case of the Varian Millennium MLC, the leaf nut is also sacrificial in that it is designed to break upon leaf collision so that the leaves themselves and other parts of the drive train are not damaged.

Machine log files, also known as DynaLog files for the Varian C‐series linac, are a record of the dose fraction, gantry angle, and MLC positions as a function of time during dynamic treatments as recorded by the linac.^18^ The MLC log file data is generated from the MLC controller, which records the MLC motor counts and then calculates leaf position by application of a conversion factor.^19^ Due to their convenience and high temporal resolution, the use of machine log files as input data for linac and patient‐specific QA has been heavily investigated.^19^, ^20^, ^21^, ^22^, ^23^, ^24^, ^25^, ^26^, ^27^, ^28^, ^29^, ^30^, ^31^, ^32^, ^33^, ^34^, ^35^, ^36^, ^37^, ^38^, ^39^, ^40^, ^41^, ^42^, ^43^, ^44^, ^45^, ^46^, ^47^, ^48^, ^49^, ^50^, ^51^, ^52^, ^53^, ^54^, ^55^ Log file–based patient‐specific QA usually involves the recalculation of the treatment plan with control points modified based upon log file data used as a representation of the “actual” delivery to be compared to the planned delivery.^23^, ^24^, ^25^, ^26^, ^27^, ^28^, ^32^, ^34^, ^35^, ^36^, ^41^, ^42^, ^43^, ^45^, ^46^, ^47^, ^50^, ^51^, ^52^, ^55^ A number of authors have cautioned about the nonindependence of log files from the systems under investigation and potential insensitivity of log files to MLC mis‐calibration or to faults in the MLC drive train and hence have suggested a need for separate MLC QA to assure log file accuracy.^20^, ^22^, ^23^, ^32^, ^37^, ^38^, ^43^, ^45^, ^50^ In an attempt to validate log file–based QA systems, a number of authors have attempted to prove the sensitivity of log file–based QA to MLC position errors via a modification of treatment plan MLC positions to simulate leaf mispositioning.^32^, ^41^, ^42^, ^48^, ^50^, ^55^, ^56^ However, it has been demonstrated via an electronic portal imaging device (EPID) imaging that log files can be insensitive to certain MLC positioning errors.^31^, ^40^, ^54^ In the study of Agnew et al.,^31^ MLC position errors detected by the picket fence test were not evident in the log files. The errors were resolved when the MLC leaf motor or T‐nut was replaced. Similar findings were made by Neal et al.,^40^ and Lim et al.,^54^ when investigating dynamic treatment delivery using cine‐EPID imaging techniques. To the authors’ best knowledge, the study of Katsuta et al.,^38^ is the only study to date that addresses such log file inaccuracies as an uncertainty rather than in a binary manner, whereby log files are either considered validated or not. In Katsuta et al.’s study, the clinical significance of log file insensitivity to a 0.5‐mm systematic MLC error was calculated to be 1.6% for target volumes and 1.1 Gy for organs at risk for the patient cohort that they investigated.

The three studies that have revealed log file insensitivities to certain MLC position errors all detected the errors via chance encounters.^31^, ^40^, ^54^ As such, the type and magnitude of errors and confounding variables were not controlled. It is the primary aim of this study to simulate the T‐nut wear caused by extensive repeated use in the clinic that has the effect of introducing backlash into MLC motions. This is achieved via a controlled experiment such that both the sources of errors are known, and multiple magnitudes of errors can be set at meaningful levels so that the sensitivity of log files, cine‐EPID‐based imaging of dynamic plan delivery and standard routine EPID‐based MLC test procedures can be assessed. It is hypothesized that EPID‐based measurements will be sensitive to the backlash‐induced errors, whereas the log files will not. For Varian linacs, MLC leaf backlash is minimized via the use of a built‐in engineering backlash test with T‐nut replacement recommended for any leaf where the backlash exceeds 0.4 mm at isocenter. If this tolerance is breached, the procedure is for Varian service personnel to replace T‐nuts. However, at this threshold, the system does not prevent clinical use but simply provides a warning during the initialization process. As such, the secondary aim of this study is to inform on the behavior of MLC leaf positioning during dynamic treatment delivery when realistic levels of MLC leaf backlash are present.

## 2 METHODS

### 2.1 Materials

Measurements for this study were primarily performed on a single Varian Trilogy C‐series linac with Millennium 120 MLC software version 8.1 immediately before it was decommissioned. A reduced version of the experiment was performed on a Varian TrueBeam linac with Millennium 120 MLC running software version 2.7 Maintenance Release 4. During all dynamic deliveries used in this study log files were recorded, and the MLC positions from these were assessed. For the Varian C‐series linacs, log files are recorded every 50 ms.

#### 2.1.1 Built‐in MLC leaf backlash test

The MLC backlash test that is available with the Varian linac for servicing and used in this study is from the Varian MLC service tool V3.0. The test operates by moving each MLC leaf individually into position to break the infrared beam and then recording the number of motor counts required to move the leaf in the opposite direction to clear the beam. The process is repeated multiple times with the results averaged and converted into a distance, which equates to the amount of backlash reported by the test. A 0.4‐mm threshold at isocenter is set for the test after which T‐nut replacement is to be performed. This threshold is consistent between the C‐series and TrueBeam linac types. For the purposes of the experiment, the test was set to the default of reporting on the average of four repetitions. Five repetitions were attempted to align the number of measurement repeats with the other test methodologies used in this study, but this caused the software to crash so was not pursued.

#### 2.1.2 Micrometer

The micrometer used for physical distance measurements in the study was an RSPro 50‐700‐001 (RS Components, Sydney, NSW, Australia), which is rated to an accuracy of ± 0.003 mm and a repeatability of ± 0.001 mm (RSPro 50‐700‐001 data manual). The micrometer was zeroed immediately prior to measurements, and the accuracy of the zeroing was checked periodically throughout the measurement session.

#### 2.1.3 Routine MLC leaf position QA test (Stakitt test)

The EPID‐based MLC leaf position tests in this study included the departmental routine monthly MLC positioning QA based on methodology modified from the method of Bayouth et al.,^57^ translated onto EPID. This modified method is known locally as the “Stakitt” test, where “Stakitt” is the Norwegian for “Picket.” The method utilizes a step‐and‐shoot test field, including 6 × 2‐cm^2^ wide strip fields separated in turn by 2 cm, which by necessity and design require a carriage shift after the third strip to be delivered. On a Varian linac, the range of MLC leaf travel can be extended by shifting the whole MLC bank via the translation of the carriage in which the MLC leaves are housed. This is known as a carriage shift and can be performed mid‐beam during large field intensity‐modulated radiation therapy beam delivery, but it is not available for volumetric‐modulated arc therapy (VMAT) type deliveries. During a carriage shift, the beam is held until the carriage and leaves are in the correct position. The Stakitt delivery has been purposely designed to test the MLC leaf position accuracy following a carriage shift.

**FIGURE 2:**
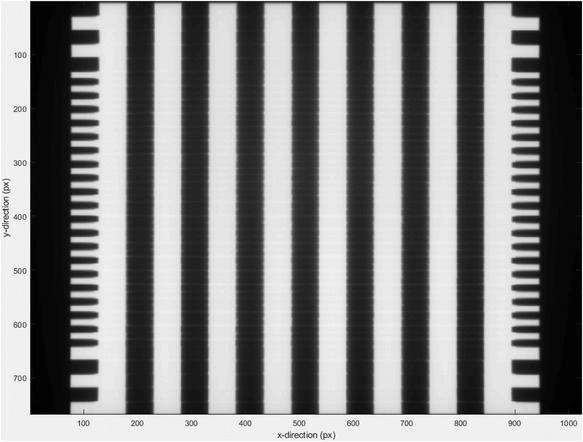
Example Stakitt test plan image.

A Stakitt test image example is provided in Figure 2. On the outermost pickets at both sides of the image, every second MLC leaf is extended so as to create a saw‐tooth pattern. A profile through this pattern is used to locate each MLC leaf and when compared to the opposing side of the image, each MLC leaf pair's trajectories can be determined irrespective of any rotations in the treatment head collimator or EPID panel and hence any such rotations are effectively accounted for in the analysis. The spatial reference point is set in the EPID coordinates from a pair of jaw defined 10 × 10‐cm^2^ field images delivered to the EPID at collimator angles of 90 and 270 degrees acquired in the same Stakitt measurement session. The center‐of‐field averaged between these two images equates to the collimator rotation axis. On the inner pickets, the MLC leaf position is found for each leaf from both banks in each strip field as the 50% grayscale value. The measured leaf position is compared to the measured collimator rotation axis position to provide a distance result that is then compared to the planned distance after accounting for the leaf offset.

#### 2.1.4 Dynamic MLC QA test (QuAVER test)

The other EPID‐based MLC test utilized in the study is the “QuAVER” methodology of Zwan et al.,^56^ which provides dynamic MLC positioning evaluation throughout the delivery using cine‐EPID images. On each cine‐EPID frame, the MLC position is measured using the 50% grayscale value and compared to the collimator rotation axis, which in the case of QuAVER has been determined per gantry angle to account for EPID panel sag. Each frame is also tagged with the gantry angle that it was acquired at using data from the CBCT encoder. The CBCT encoder is used for this purpose rather than the readout potentiometers due to the encoder's superior readout resolution. Measured MLC positions in each frame versus gantry angle are compared against the expected positions of the plan. The methodology provides a time/gantry‐resolved measurement of the dynamic plan delivery. For this study, the dynamic VMAT plans used for testing were identical to the original QuAVER study,^56^ and the reader is referred to this study for details of the plans. Plans selected for testing included the VMAT test plan designed to fully stress test the MLC under its full range of allowed motions, gravitational conditions, and friction due to interdigitation (Plan 8: MLC Test 2: Interdigitating sliding window during gantry rotation^56^). This plan is in the form of an oscillating sweeping gap with adjacent leaves offset from one another so as to interdigitate and hence include friction between leaves. MLC leaves are moved throughout the plan across the full range of allowed speeds. The plan is delivered with a collimator angle of 0 degrees so that leaf travel is fully with and against gravity when the arc traverses gantry angles 90 and 270 degrees. Measurements were also performed using a clinical VMAT head‐and‐neck treatment plan (Plan 9: MLC Test 3: clinical VMAT delivery^56^). This plan was chosen to provide measurement in a representative clinical scenario.

### 2.2 Measurement methods

#### 2.2.1 Data acquisition

Measurements were primarily performed in a single session immediately prior to linac decommissioning. The experiment followed the following steps:

1. Six of the even numbered inner MLC leaves (0.5‐cm width) from each carriage were selected for modification (Table 1). Even numbered leaves were selected for ease of access to the T‐nut. Leaves were chosen so as not to be adjacent to other modified leaves or interdigitate with modified leaves from the opposing bank. These selections were chosen to avoid interactions between modified leaves.
2. For the selected leaves the T‐nuts were replaced with new ones so as to minimize initial backlash.
3. The MLC was initialized after which the MLC built‐in backlash measurement procedure was performed with the standard four repetitions. Results were recorded.
4. The Stakitt test was performed five successive times each at gantry 0, 90, and 270 degrees (International Electrotechnical Commission convention) with these gantry angles selected to represent the standard G0 (treatment head pointing toward the floor) plus the cardinal angles in which gravity acts most with and against MLC motion. Log files were recorded for each delivery.
5. The QuAVER test was performed five successive times for both the representative clinical head‐and‐neck plan delivery and the test plan designed to provide the maximum stress on the MLC. Log files were recorded for each delivery.
6. The selected T‐nuts were then removed from the MLC, and their lengths were individually measured via a micrometer. The top of the “T” was then sandpapered down to introduce a mechanical play of the T‐nut in the leaf housing to produce a similar effect to that which occurs when T‐nuts wear (see Figure 1). The magnitudes of the length reductions, as measured by the micrometer, were set to approximately 0.2 mm for three of the T‐nuts in each bank and approximately 0.3 mm for the other three in each bank. These magnitudes were chosen to introduce theoretical leaf position errors approaching the recommended clinical tolerances (0.5 and 1.0 mm) when added to the initial backlash inherent to the new T‐nuts and translated to isocenter plane. Due to the imprecise nature of T‐nut length adjustments via the sand paper, a range of adjustments were effected within this range across the modified leaves.
7. The MLC was reinitialized and the backlash test repeated with changes to the backlash on the modified leaves recorded.
8. The Stakitt and QuAVER measurements were repeated five times with MLC initialization updated between each measurement set. Log files were recorded for all deliveries.

**TABLE 1:** Measured backlash and micrometer results for the modified leaves

	Distance at isocenter plane (mm)			
MLC leaf	Total backlash after	Backlash change	Micrometer change	Difference micrometer change—backlash change
A20	0.55	0.31	0.39	0.08
A24	0.76	0.57	0.56	−0.01
A28	0.59	0.33	0.40	0.07
A34	0.78	0.67	0.62	−0.05
A38	0.74	0.59	0.60	0.01
A42	0.57	0.37	0.49	0.12
B22	0.71	0.49	0.46	−0.03
B26	0.80	0.59	0.63	0.04
B30	0.67	0.51	0.44	−0.07
B32	0.86	0.61	0.66	0.05
B36	0.67	0.43	0.46	0.03
B40	0.80	0.55	0.60	0.05

^a^Multi‐leaf‐collimator.

A subset of the experiment was also performed on a TrueBeam type linac with Millennium MLC to investigate the applicability of findings to the TrueBeam system. In this secondary experiment, a single leaf was modified to a magnitude consistent with the primary experiment using the same procedure. The MLC was re‐initialized after leaf modification and the built‐in backlash measured after modification was compared to before modification.

#### 2.2.2 Data analysis

##### Micrometer and built‐in backlash test data

The measured change in the backlash from the built‐in test was compared to the change in T‐nut length as measured by the micrometer after the conversion of the micrometer‐measured distances from the MLC leaf plane to the isocenter plane.

##### Routine MLC position QA test (Stakitt test)

For the five initial Stakitt measurements, the standard deviation for each leaf in each picket was calculated as a measure of repeatability. The measured difference before and after T‐nut modification was calculated for all five measurements. The picket immediately after the carriage shift was removed from analysis for reasons that will be provided in Sections 3 and 4 meaning that five MLC positions (pickets) per leaf were included per measurement resulting in 25 samples per leaf (5 measurements × 5 pickets). The measured deviations after modification minus before modification were calculated for each sample. Analysis was similarly performed for a control group comprising the average of five control leaves for which no T‐nut modification had been made. The process was repeated for the log file data acquired with the Stakitt deliveries with analysis performed using Matlab V2019a (MathWorks, Natick, MA, USA). The Stakitt measured difference before and after modification was compared for the modified leaves and the control group relative to the mean of the control group.

##### Dynamic treatment delivery tests (QuAVER)

For each arc in each test plan the results were post processed to provide MLC positions every 0.1 degree gantry rotation. This allowed the five measurements pre‐modification and the five measurements post‐modification to each be averaged. The standard deviation of the pre‐modification measurements was used to assess repeatability. Due to known inaccuracies in the tagging of gantry angles to each EPID frame,^58^ the trajectories were shifted in gantry angle by 0.8 degrees to provide synchronization via the first control point. The resulting mean MLC trajectories were compared to the plan. Similar analysis was applied to the log file data.

## 3 RESULTS

### 3.1 Micrometer and built‐in backlash test

The deviation between the mean measured change in backlash compared to the mean measured change in micrometer distance across all modified leaves was 0.02 ± 0.06 mm (1 SD, Table 1). This result strongly suggests that the amount of measured backlash equals the micrometer measured T‐nut change.

### 3.2 Routine MLC position QA test (Stakitt test)

#### 3.2.1 Log files from Stakitt deliveries

The log files acquired during Stakitt deliveries were analyzed for sensitivity to MLC leaf position variation caused by additional backlash from the modified T‐nuts. The difference in MLC log file recorded positions across all of the modified and control leaves across the three gantry angles at which measurements were taken before and after T‐nut modification was consistent with a mean value of 0.00± 0.01 mm (1 SD) at an isocenter plane.

#### 3.2.2 Stakitt EPID‐based measurements

The standard deviation for the Stakitt measurements for each leaf across all five pickets for the five initial measurements was calculated to range between 0.00 and 0.06 mm. As such, the 0.06 mm maximum is considered the Stakitt measurement repeatability.

**FIGURE 3:**
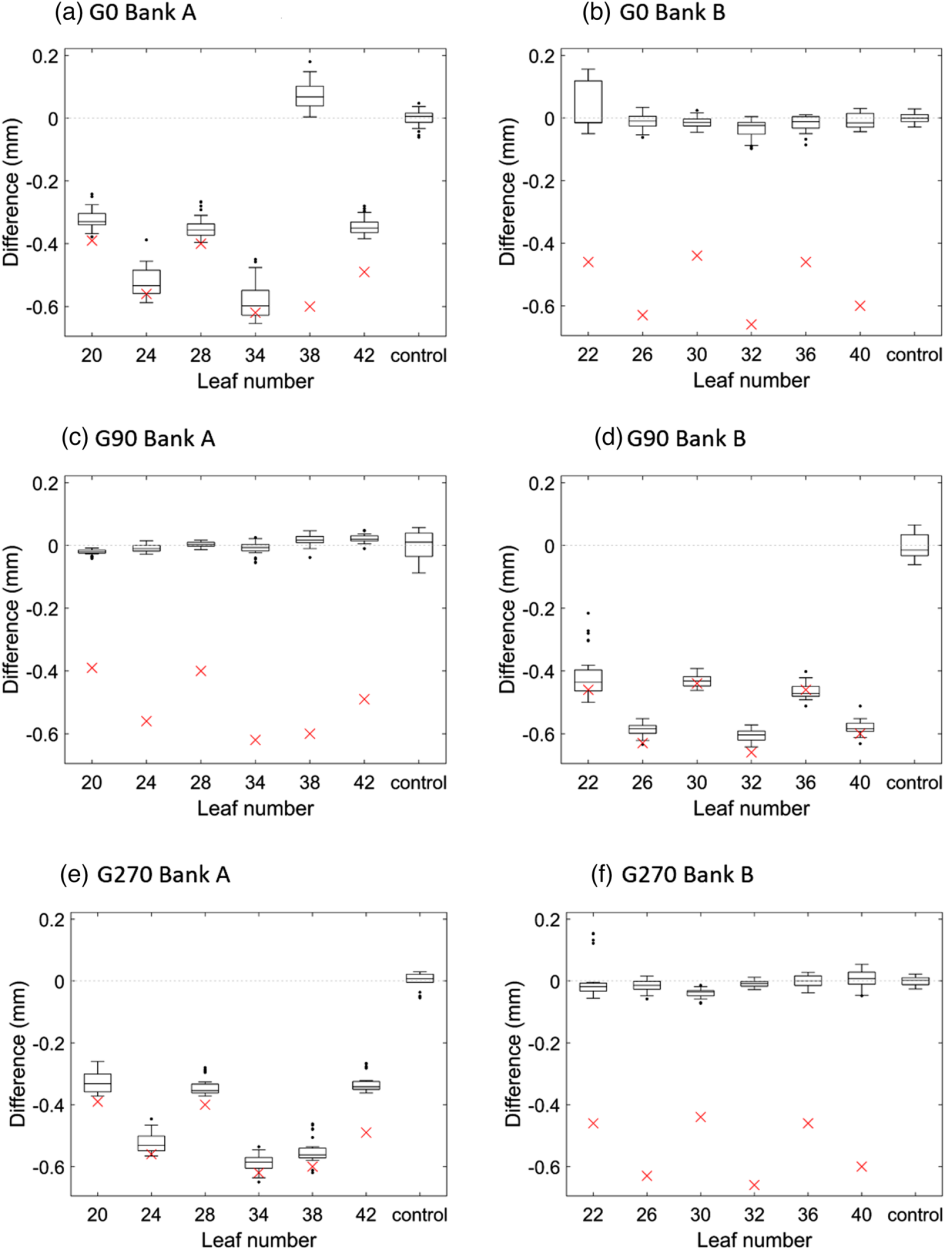
Stakitt test measured results. Difference (mm) between before and after modification for each of the modified leaves alongside a combined set of five unmodified control leaves. Results normalized to the mean of the control leaves. The boxplot whiskers cover 95% of the data with any outliers plotted individually. Differences measured by the micrometer included (red crosses) for comparison. Differences between the mean Stakitt measured value and the micrometer result presented in the accompanying tables for each leaf. a) Gantry 0 degrees for MLC bank A, b) Gantry 0 degrees for MLC Bank B, c) Gantry 90 degrees for MLC Bank A, d) Gantry 90 degrees for MLC bank B, e) Gantry 270 degrees for MLC Bank A, f) Gantry 270 degrees for MLC Bank B

The results of Figure 3a generally show that for each modified leaf the Stakitt test has detected a change of similar magnitude to the micrometer change. This suggests that the full magnitude of leaf backlash has translated into position error of the leaf and that Stakitt is sensitive to the error. Leaf 38 on Bank A (A38) is a clear outlier with the measured leaf backlash after modification improved to the order of 0.1 mm. This was unexpected and it is hypothesized that when the leaf A38 T‐nut was reinserted into the leaf after modification, it either jammed into the leaf recess or grit from the sandpapering may have lodged in the T‐nut screw thread and either way there was a jamming effect and backlash was actually reduced. For the other five modified leaves, the measured deviation between Stakitt and micrometer averaged 0.07 ± 0.05 mm (1 SD) that is of order of the Stakitt measurement repeatability and an order of magnitude less than the measured distances. In Figure 3a, leaf 42 exhibits a reduced Stakitt measured position change compared to that expected from the micrometer change in comparison to the other modified leaves. This could be explained by increased friction between this leaf and its neighbors relative to the other modified leaves resulting in less of the available backlash being taken up and translating into position errors. The levels of agreement observed between Stakitt and micrometer are considered well within clinical acceptability and suggest that the Stakitt test is sufficiently sensitive to realistic levels of MLC leaf backlash.

As opposed to Bank A, the results of Figure 3b for Bank B at Gantry 0 degrees (G0) indicate that none of the introduced backlash has translated into position error in the modified leaves. This discrepancy between Banks A and B is hypothesized to be due to the fact that one bank in the measurement is pushing the leaves into position, whereas the other is pulling the leaves. Depending on the direction in which the leaves were moved into their initial position for measurement, the backlash would have either been fully taken up or the opposite, and this would then translate into the measured positions as observed. This result is expected and the reason why leaves were modified in both banks was to demonstrate this phenomenon. To assist with explaining this, the theoretical leaf motions when backlash is present are provided in the Supporting Information. The Stakitt results of Figure 3a,b demonstrate agreement with these theoretical motions.

The MLC motions presented in the Supporting Information apply to G0, which is the scenario when gravity is perpendicular to leaf motion. When the gantry is rotated to G90 as per Figure 3c,d, gravity works in the direction parallel to leaf motion and hence influences how the backlash translates into position error. The Bank A and B results of Figure 3c,d are reversed from Figure 3a,b. At G90, gravity is forcing the leaves in Bank A back into the carriage, which is below the leaves and hence all of the backlash is taken up resulting in no measured change being observed in leaf position before and after modification. Interestingly, the A38 leaf that was an outlier at G0 appears at G90 to behave consistently with the other modified leaves, suggesting that if the T‐nut got stuck in the leaf recess at G0, then it potentially was freed with the assistance of gravity. For Bank B where the carriage is above the leaf at G90 gravity forces, the leaf out of the carriage and hence the full backlash is translated into position error. For Bank B at G90, the mean deviation between Stakitt and the micrometer‐measured‐introduced backlash is 0.03± 0.02 mm (1 SD), which is within the Stakitt measurement repeatability.

The results of Figure 3e,f are inverted opposite of Figure 3c,d. This is expected with the hypothesized effect due to gravity and considering that at G270, the MLC banks are reversed with respect to gravity compared to G90. At G270, the mean deviation between Stakitt and micrometer is 0.07± 0.05 mm (1 SD), which is identical to the G0 result and of similar magnitude to the Stakitt measurement repeatability.

**FIGURE 4:**
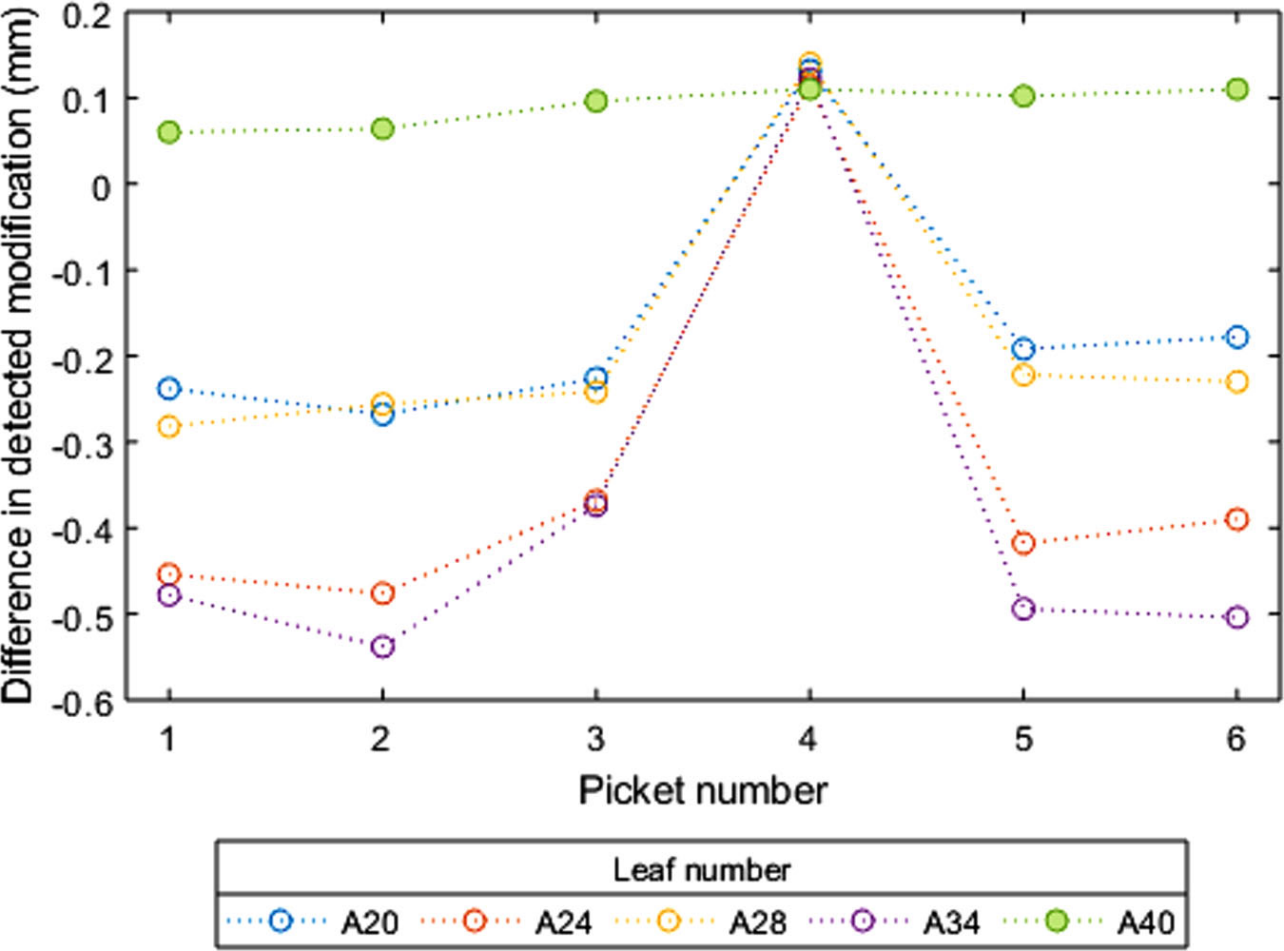
Stakitt measured leaf position differences before/after modification for each of the modified leaves plotted per picket number. A40 is included as an example control leaf.

The data for Picket 4, which is the picket immediately after the carriage shift in the Stakitt test plan, was removed from the analysis so far presented. Figure 4 presents the measured position difference before/after modification for the modified Bank A leaves excepting the outlier leaf A38 presented per picket.

Figure 4 shows that within Stakitt repeatability, the difference before/after modification for each of the modified Bank A leaves was consistent between pickets. The exception is at Picket 4, which is immediately after the carriage shift. Figure 4 shows that at Picket 4, the position error due to the added backlash has been removed, and the difference of all modified leaves has reverted to that of the control leaf. During a carriage shift, the MLC leaves are retracted or extended to compensate for the movement of the carriage. This motion is in the opposite direction to the leaf motion throughout the rest of the delivery and has the effect of reversing whether the backlash is fully taken up or none taken up as per the theoretical motions presented in the Supporting Information. When the leaves revert to their original direction of motion for Picket 5, the previous behavior is observed.

### 3.3 Dynamic treatment delivery tests (QuAVER)

For the five deliveries pre‐modification, the QuAVER repeatability was measured to be ± 0.03 mm (1 SD). After averaging both the five test plan QuAVER measurements pre‐modification and the five test plan QuAVER measurements post‐modification, the mean deviation for each modified leaf was calculated across the leaf's full trajectory for both arcs. These results are presented in Table 2 with comparison to the built‐in backlash test results. The results for the clinical plan (Plan 9) were very similar to those of the test plan (Plan 8), so for the purposes of brevity, only the Plan 9 results are presented. Based upon the differences observed with gantry angle from the Stakitt analysis, the post‐modification QuAVER results have also been presented in Table 2 split between positive (G0–G180) and negative (G360–G180) gantry angles and combined across all gantry angles.

**TABLE 2:** Mean QuAVER measured multi‐leaf‐collimator (MLC) deviations from plan for the test plan compared to the built‐in backlash test results

		Measured mean leaf position error (abs, mm, both arcs combined)							
		Before T‐nut modification		After T‐nut modification					
Bank	Leaf	Measurement: all gantry angles	Difference (measurement—backlash)	Measurement: all gantry angles	Difference (measurement—backlash)	Measurement: negative gantry angles	Difference (measurement—backlash)	Measurement: positive gantry angles	Difference (measurement—backlash)
A	20	0.28	0.04	0.39	−0.16	0.60	0.05	0.20	−0.35
	24	0.12	−0.08	0.39	−0.37	0.71	−0.06	0.07	−0.69
	28	0.17	−0.09	0.23	−0.35	0.40	−0.19	0.07	−0.52
	34	0.00	−0.12	0.23	−0.56	0.60	−0.18	0.14	−0.65
	38	0.05	−0.11	0.25	−0.50	0.57	−0.18	0.07	−0.67
	42	0.05	−0.14	0.03	−0.54	0.31	−0.26	0.24	−0.33
B	22	0.19	−0.02	0.02	−0.68	0.12	−0.58	0.17	−0.54
	26	0.07	−0.15	0.26	−0.54	0.03	−0.77	0.55	−0.25
	30	0.03	−0.13	0.22	−0.44	0.04	−0.63	0.43	−0.24
	32	0.06	−0.19	0.35	−0.51	0.01	−0.85	0.69	−0.17
	36	0.02	−0.22	0.23	−0.43	0.08	−0.58	0.54	−0.13
	40	0.13	−0.12	0.43	−0.37	0.03	−0.77	0.76	−0.05

From the results of Table 2, prior to T‐nut modification, the QuAVER‐measured mean deviation from the plan for all leaves along the entire trajectory for both arcs in Test Plan 9 was 0.1± 0.08 mm (1 SD). The mean deviation when compared to the built‐in backlash test was −0.11 ± 0.07 mm (1 SD). After the T‐nut modification, the mean deviation between the QuAVER measurements and the backlash test was 0.46 ± 0.13 mm. For all leaves except A20, the mean deviation equated to approximately half or less of the magnitude of the T‐nut modification. This is expected in the context of the Stakitt results where, with the gantry on one side, the full backlash is translated into leaf position error and on the other side none of it is translated. In the context of arc deliveries like those performed for QuAVER, the mean errors over the full arc would then be expected to be approximately half of the introduced backlash as is observed. This is borne out in Table 2 results where for the negative gantry angles, the mean Bank A deviation from the backlash test was −0.14 ± 0.11 mm (1 SD) for Bank A and increased in magnitude to −0.70 ± 0.12 mm (1 SD) for Bank B. However, for the positive gantry angles, the effect is reversed with a mean deviation from the backlash test for Bank A of −0.54 ± 0.16 mm (1 SD) and for Bank B of −0.23 ± 0.17 mm (1 SD). The consistent negative deviation between QuAVER measurements and the backlash suggests that not all of the backlash was translated into position error, which is potentially due to friction between leaves.

**FIGURE 5:**
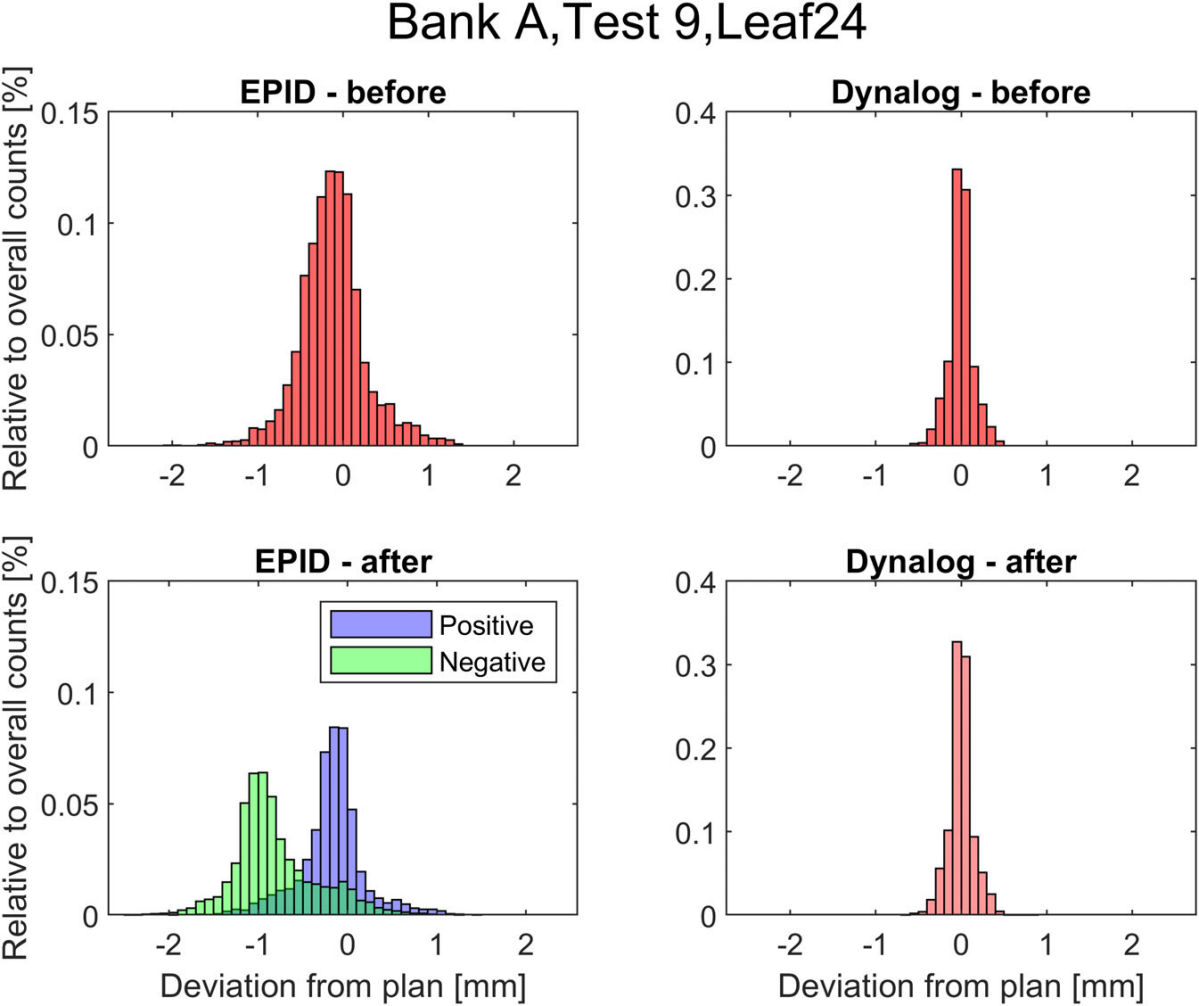
QuAVER result histograms for measured deviations to the plan for Leaf A24 for Test Plan 9, including both arcs. Top left = electronic portal imaging device (EPID) measurements before T‐nut modification, top right = log file measurement before T‐nut modification, bottom left = EPID measurement after modification, bottom right = log file measurement after modification. The EPID‐after histogram has been presented split into different colors for the positive gantry angles (toward G90) and the negative gantry angles (toward G270).

The results in Figure 5 are representative of all modified leaves and for both Test Plans 8 and 9. Before modification, both EPID and log file distributions are centered about 0‐mm deviation (EPID mean deviation: −0.12 mm, log file mean deviation: 0.00 mm) with a greater spread in the EPID data (EPID: ± 0.4 mm [1 SD] and log file: ± 0.14 mm [1 SD]). The increased spread associated with the EPID measurement may be caused by uncertainties in the EPID measurement and/or may be due to the insensitivity of the log files, including to the initial inherent backlash. After modification, the log file distribution is virtually identical to before modification (0.00 ± 0.14 mm [1 SD]) providing strong evidence of the log file insensitivity to the T‐nut modification. However, after modification, the EPID distribution has changed with two peaks now apparent that correspond to gantry angles either side of zero. This result is in agreement with the Stakitt findings and the global QuAVER findings presented in Table 2. The positive gantry angle distribution is centered on zero deviation with mean deviation and spread similar to that pre‐modification (−0.07 ± 0.37 mm [1 SD]). However, the negative gantry angles distribution is offset (−0.71 ± 0.48 mm [1 SD]). This finding is apparent on all modified leaves with the peaks offset by a magnitude similar to the T‐nut modification for each particular leaf.

**FIGURE 6:**
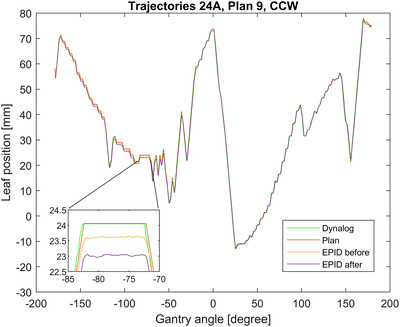
Multi‐leaf‐collimator (MLC) leaf A24 trajectory during CCW arc in Test Plan 9 as measured with QuAVER electronic portal imaging device (EPID) before and after T‐nut modification and with log file.

The results of Figure 5 can be visualized via Figure 6, which presents the trajectory of MLC leaf A24 during the Counter Clockwise (CCW) arc of Test Plan 9. For comparison purposes, the EPID measurements before and after T‐nut modification are plotted against the plan and the log file data. The log file data is virtually indistinguishable before and after modification, so only the after data is included.

The MLC trajectory presented in Figure 6 demonstrates the high level of agreement both before and after T‐nut modification to the plan for both the EPID and log file data for leaves of Bank A for the positive gantry angles. Figure 6 also demonstrates the deviation of the EPID data for the negative gantry angles due to the backlash. This is most clearly shown in the inset that is zoomed in on the trajectory between gantry angles −70 to −85 degrees. In this inset, the log file–recorded MLC position qualitatively matches the plan except for a slight delay. However, the EPID‐measured position is offset for the before modification measurement by approximately 0.5 mm and then for the post‐modification measurement by approximately 1 mm. To put this into context, what is expected, as per Table 1, is the backlash measured by the built‐in test for this leaf which was measured to be 0.2 mm pre‐modification and 0.76 mm after‐modification.

Because it was an outlier in the G0 Stakitt results of Figure 3, the leaf A38 trajectory in the QuAVER data was specifically chosen for investigation. Leaf A38 was found to show similar behavior to the other leaves which had introduced backlash except that the divergence between measurement and plan that is typically observed at G0 for these leaves was observed at approximately G‐22 (G338). This fits with the Stakitt results and is why this leaf is an outlier in the G0 Stakitt plot.

For the secondary experiment performed on the TrueBeam type linac, the backlash before leaf modification was measured to be 0.59 mm (converted to isocenter plane). The T‐nut length was reduced via sandpapering by 0.26 mm as measured with the micrometer and converted to isocenter plane, and the backlash after modification was measured to be 0.96 mm at isocenter. The measured change in backlash agreed with the micrometer change to within 0.11 mm, which is comparable to the results of the main experiment as presented in Table 1. The magnitude of the final backlash is slightly larger than any from the main experiment. Similar to the C‐series, the TrueBeam MLC initialization process provided a warning but did not fail at this magnitude of backlash.

## 4 DISCUSSION

Log files have been demonstrated to be insensitive to backlash‐induced MLC position errors of magnitude approaching clinical tolerances and for which the linac did not prevent treatment delivery. These findings are in agreement with and build upon those from previous studies.^31^, ^40^, ^54^ Such insensitivity creates a significant risk of false indication of acceptable plan deliverability results from log file–based patient‐specific QA methods. Although the experiments of this study were performed primarily on a C‐series type linac, the results of the secondary experiment indicate that results are likely applicable to the TrueBeam linac also. It is uncertain whether the findings apply to other MLC types such as those utilized in the Varian Halcyon or Elekta (Elekta Inc, Stockholm, Sweden) linac systems. However, any MLC system that utilizes T‐nuts or other non‐perfectly rigid mechanical separation in the drive train between leaf motor and leaf is potentially susceptible to backlash‐introduced leaf positional error that may not be captured by log files, the data of which is recorded from the MLC motors.

Considering that with regards to MLC, log files are simply records of the MLC motor counts and that there is mechanical separation between the motor and the MLC leaf then insensitivity to MLC leaf backlash of log files is theoretically expected. Log files are hence inherently inaccurate up to the order of the backlash present in the MLC leaf. This is a good example of the risk associated with using records produced by the MLC control system to then test the very same MLC and is why measurement independence from the systems under investigation is a fundamental QA principle.

The associated leaf positioning inaccuracy due to MLC leaf backlash can be minimized with strict control procedures such as tight tolerances applied to the built‐in MLC backlash procedure. However, reducing backlash to zero is mechanically infeasible and as per current procedures, MLC log file data should be considered having significant uncertainty as high as 0.4 mm at isocenter based upon the current level at which T‐nut replacement is recommended. It should be cautioned that the backlash test frequency is currently set in the vendor maintenance procedures to be performed every 240 days on the TrueBeam system and every 120 days on the C‐series, so actual backlash may be up to or outside the 0.4‐mm range for an extended period. This low frequency of testing could be mitigated procedurally by reviewing the backlash results in the MLC initialization procedure. Within MLC initialization, it was found that if backlash was measured outside the threshold then this was flagged to the user with a recommendation for service, but that clinical treatment was not prevented.

A similar way to reduce uncertainty in log files due to backlash could potentially be via regular MLC position QA. This would require the MLC QA to have high accuracy to allow tolerances set tighter than the built‐in backlash test. However, such QA testing should not be thought of as log file validation as it at best can still only ensure that log files are accurate to the tolerance of the QA test, and hence this tolerance should still be the uncertainty level applied to subsequent MLC positions used for QA based upon log file data.

The concept of log file validation via independent measurement is flawed without sensitivity testing because the purpose of QA is to detect clinically significant suboptimal system performance. As such, obtaining similar results between log file data and independent measurement while the machine is performing well does not constitute validation. Validation occurs when sensitivity of a QA test to real‐world failure modes is proven. It is unclear from the literature to what MLC failure modes log files have had demonstrated sensitivity. Also, such sensitivity is not demonstrated by changing MLC positions in plans to simulate MLC errors and then comparing the log files from the original plan to the modified plan. In such simulations, no failure mode of the MLC is actually introduced and all that is demonstrated is that log files are sensitive to slightly different plans rather than to MLC error or suboptimal performance.

Having dynamic measurements to test dynamic treatment deliveries is logical. However, the lack of independence from the MLC system and demonstrated insensitivities of log files make them a nonideal data source for such QA. Cine‐EPID imaging has been evaluated for this purpose.^54^, ^56^, ^59^ Although EPID imaging has its own inherent inaccuracies and uncertainties, it is at least independent of the MLC, gantry and dose control systems used to modulate a VMAT delivery if not from the linac itself, whereas log files are not independent of the dynamic systems under investigation. Cine‐EPID imaging has been demonstrated as a data source for plan recalculation to allow DVH analysis of actual plan deliveries,^59^ and it has also been recently claimed that the process has been developed to the required accuracy that it can be used to validate log files.^54^ If this level of accuracy is confirmed then cine‐EPID could be considered to be a superior patient‐specific QA data source to log files as it is free of MLC drive train error insensitivity and is independent of the systems under investigation while maintaining the level of required accuracy and convenience. Of course, an extra layer of information would be added to testing if log file data were included alongside cine‐EPID data and both are presented against the planned delivery to provide both an independent measurement of the delivery and the linac's record of the delivery. It is suggested that linac vendors improve their cine‐EPID imaging capability to allow for such comparison, so that cine‐EPID methodologies alongside log files such as those previously published^54^, ^56^, ^59^ can be adopted and be further developed and evaluated for mainstream clinical use.

It is recognized that the sandpapering of the “T” on the T‐nut to introduce backlash may not necessarily represent how a T‐nut naturally wears with use. However, it is known that T‐nuts wear over time and introduce backlash into the MLC leaf tip. This is the reason why the built‐in MLC leaf backlash test exists in the Varian system. Although the failure mode may not in reality perfectly replicate the true situation, the point of the experiment was to introduce backlash in a relatively controlled manner to predefined magnitudes while being able to maintain consistency of other variables.

The MLC motions recorded in this study when MLC leaf backlash is present show that whether the backlash translates into position error is heavily dependent on gantry angle and the direction from which the MLC moved into its start position prior to delivery. Gantry angle dependence of MLC positions has been demonstrated previously in log file data,^60^ but to much smaller magnitudes than are presented in this current study, which includes the significant influence of backlash. Gantry angle dependence is likely gravity related and whether gravity is pulling a leaf back out of its carriage or working to push it back in. The effect appears to result in all of the backlash translating into position error on one side of the gantry and none on the other side with the effect being reversed for the opposing MLC bank. This has potential impact on clinical plan delivery in that the MLC aperture size will be larger than planned by the amount of backlash present in the leaf in the MLC bank which is above at any point in the plan. The aperture center is also effectively shifted by half of the backlash magnitude. The results for leaf A38 show that these effects do not necessarily occur for every leaf. The results presented indicate that contrary to the other modified leaves, the position accuracy of which changed at G0 that for A38, this change occurred closer to Gantry −22 degrees (G338). It is not known why leaf A38 behaved differently, but it could be possible that the leaf has higher friction, grit from the sandpapering had gotten into the screw thread or that the T‐nut had somewhat jammed in the leaf housing such that a greater gravitational force in‐line with the leaf motion was required to initiate the change that only occurred as the gantry rotated closer to G90 or G270 degrees such that the gravity vector aligned closer to the direction of MLC motion.

The effect of carriage shifts in the MLC motions when backlash is present appears to have a resetting effect. This is logical as whether the backlash is taken up or otherwise in the leaf is dependent on the direction at which the leaf approached its start point and hence whether backlash was taken up or otherwise before plan delivery begins. Based upon this concept, when the carriage shifts the MLC motions effectively reverse the MLC motion direction and hence the behavior of the leaves from each bank are temporarily reversed.

## 5 CONCLUSIONS

Machine log files have been demonstrated to be insensitive to realistic MLC leaf backlash that produces MLC position errors of similar magnitude to published tolerances. Such inaccuracy should be minimized and the residual accounted for as uncertainty in log file–based QA. The effect of MLC leaf backlash on clinical MLC motions has been investigated, it is believed for the first time, and the effect on MLC position accuracy appears to be highly dependent on gravity and hence gantry angle with backlash either fully translating into position error or none at all.

## CONFLICT OF INTEREST

The authors declare that there is no conflict of interest that could be perceived as prejudicing the impartiality of the research reported.

## AUTHOR CONTRIBUTIONS

Michael Barnes: Developed the original idea for the study with Dennis Pomare, performed data acquisition, analysis and interpretation of results, and primarily wrote the manuscript.

Dennis Pomare: Developed the original idea for the study with Michael Barnes, performed data acquisition, and provided technical input to the study and review of the draft manuscript.

Marcus Doebrich: Processed and analyzed the QuAVER data and provided figures for results and provided scientific input into the study and review of the draft manuscript.

Therese S. Standen: Processed and analyzed the Stakitt data and provided figures for results and provided scientific input into the study and review of the draft manuscript.

Joshua Wolf: Processed and analyzed the log file data and provided scientific input into the study and review of the draft manuscript.

Peter Greer: Provided scientific input into the study and review of the draft manuscript.

John Simpson: Provided scientific input into the study and review of the draft manuscript.

## Supporting information



Supporting Information
