# Supplementary material for: Insensitivity of machine log files to MLC leaf backlash and effect of MLC backlash on clinical dynamic MLC motion: An experimental investigation
Source: J Appl Clin Med Phys. 2022 Jun 9;23(9):e13660. doi: 10.1002/acm2.13660 (PMC9512360; doi:10.1002/acm2.13660)
Supplement: Supplementary file 1 — Supporting Information [file ACM2-23-e13660-s001.docx]

**Supplementary Material**

**MLC leaf theoretical motions when leaf backlash is present.**

**In Figure 7, consider the situation of a leaf with inherent backlash that has been extended into its start position prior to field delivery so that the T-nut is resting up against the leaf in the housing and backlash has been taken up (Figure 7: Position 1). The leaf is then retracted (Figure 7: Motion between positions 1 and 2). If there is insufficient friction to overcome the inertia in the system then the leaf motor will begin to turn, the T-nut will translate, but will not immediately engage the leaf as the T-nut needs to move the distance of the backlash first within the leaf housing before it engages the leaf and thereafter begins to move it. The effect is that when the desired motor counts are achieved the leaf is still short of its expected destination by the magnitude of the backlash (Figure 7: Position 2) creating an MLC position error. If the leaf is then to be extended again (Figure 7: Motion between positions 2 and 3) then backlash needs to be taken up again before the leaf is engaged. The motor will drive the T-nut the full distance and the leaf will move by the prescribed distance minus the backlash that needed to be taken up. However, since the leaf had begun extended out from expected by the backlash then the leaf will finish at Position 3 in its expected position and there will not be any leaf position error. If the leaf is then further extended (Figure 7: Motion between positions 3 and 4) since the backlash has already been taken up at position 3 the leaf will move immediately with the motor and the leaf will move the expected distance and finish in its expected position. Finally, if the leaf is then retracted again (Figure 7: Motion between positions 4 and 5) the scenario is the same as the initial motion between positions 1 and 2 and the leaf will end up short of its destination. In this scenario, whenever the leaf is retracted a leaf position error occurs. Whenever the leaf is extended it moves to the correct position. The outcomes are reversed for the opposing bank and also if the leaf had been retracted into its start position rather than extended as per the scenario presented in Figure 7.**

**Supplementary material figure captions**

**
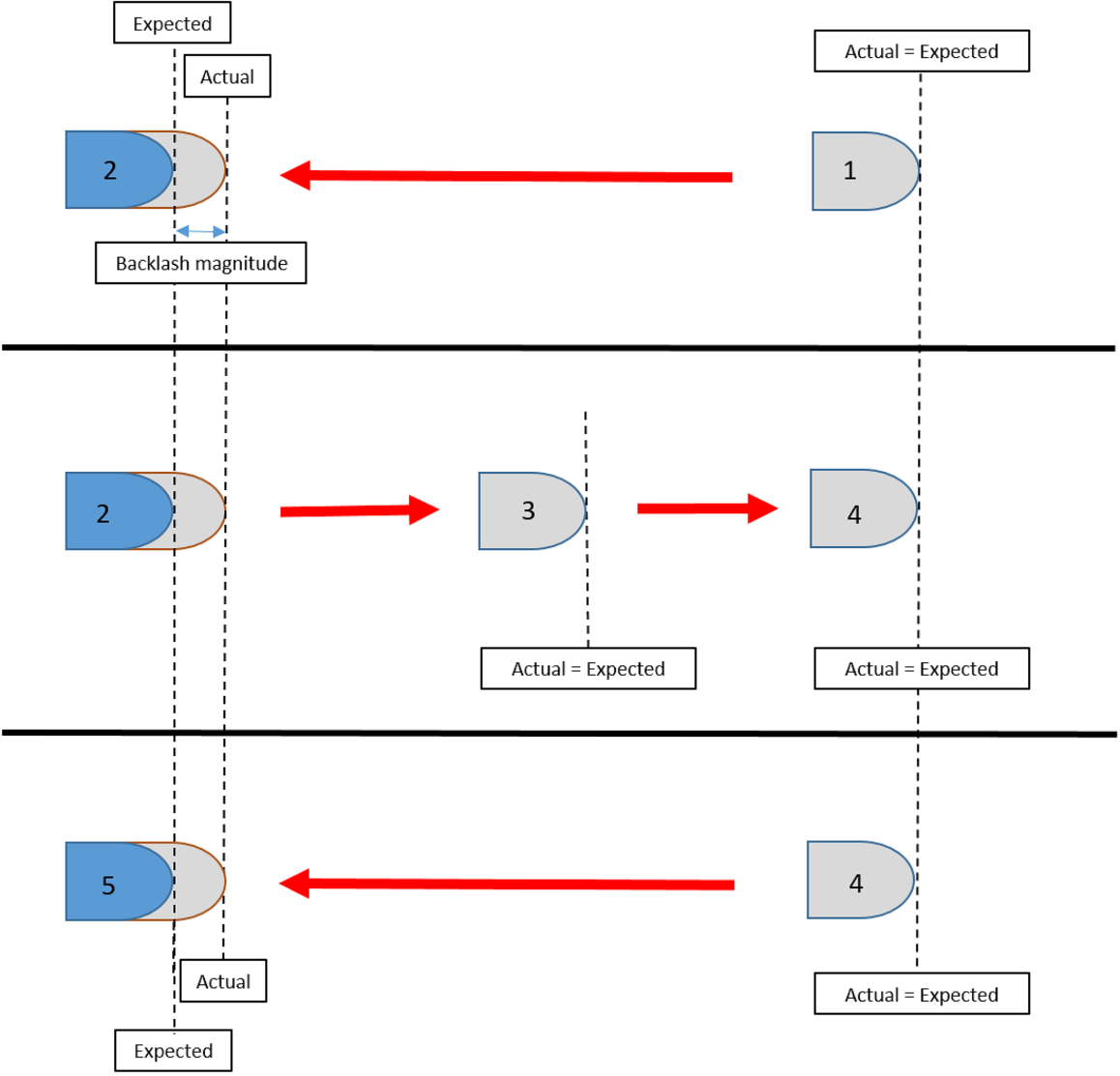
**

Figure 7 Diagram of theoretical MLC leaf motions and effect on MLC positions when backlash is present.
